# From crystal to structure with *CCP*4

**DOI:** 10.1107/S2059798317017557

**Published:** 2018-02-01

**Authors:** Michael A. Hough, Keith S. Wilson

**Affiliations:** a School of Biological Sciences, University of Essex, Wivenhoe Park, Colchester, CO4 3SQ, UK; b York Structural Biology Laboratory, University of York, Heslington, York, YO10 5DD, UK

**Keywords:** *CCP*4, structure determination, methods

## Abstract

An introduction to the 2017 CCP4 Study Weekend Special Issue.

The 2017 CCP4 Study Weekend was held at the University of Nottingham with the title *From Crystal to Structure*. The meeting covered the full process of macromolecular X-ray structure determination from indexing of the diffraction data to deposition of a fully validated structure in the Protein Data Bank. This topic was last covered at the 2010 Study Weekend and the presentations at the 2017 meeting demonstrated the many developments in CCP4 in that interval. This year’s meeting included a number of education-focused talks covering the basic principles of each stage of structure determination. CCP4 remains at the forefront of software development for macromolecular crystallography almost four decades since its founding.

The first session, *Getting the data* was opened by Martin Noble with an overview of the status of protein crystallography in 2017. This was followed by a description from Gwyndaf Evans of the newly developed *DIALS* data reduction package, with a user’s perspective of data collection and processing provided by Johan Turkenburg. The second session began with an elegant overview of experimental phasing by George Sheldrick, followed by presentations of two widely used experimental phasing approaches, *Crank*2 and *PhaserEP*, by Pavol Skubak and Randy Read, respectively.

The *Is there a homologue?* session addressed molecular replacement (MR) – now the predominant method for solving the phase problem. This was reviewed in the opening talk by Airlie McCoy, after which Ronan Keegan described the *MRBump* approach to highly automated MR. The following talks addressed options where a suitable search model is not readily available. Dan Rigden described the generation of homology models for MR using *AMPLE*, while Isabel Usón and Huw Jenkins described fragment-based approaches.

The first session of the second day covered *Refining and building*, featuring a highly interactive demonstration of model building in *Coot* from Paul Emsley. Kevin Cowtan described automation of model building and refinement, and the session was completed with a talk from Oleg Kovalevskiy on maximum-likelihood refinement. The *Did you get it right?* session addressed the critical issue of structure validation. Jane Richardson discussed the use of, and new features in, the *Molprobity* server, followed by a description of the PDB Validation Server from Sameer Velankar. Finally, Eugene Krissinel discussed the functionality and future of cloud-based CCP4 services.

The final session, *Going forwards* opened with a review of the state-of-the-art in the handling of ligands by Judit Debreczeni. Liz Potterton introduced the new *CCP*4 graphical user interface, *CCP*4*i*2. The meeting was brought to a close with a reflective talk from David Stuart, addressing the question *quo vadis crystallography*? – providing both a historical perspective and thoughts on the future of X-ray crystallography in the age of high-resolution cryo-EM and X-ray free-electron lasers.

We would like to thank all speakers and chairs for their excellent contributions together with all the authors and referees who have contributed to this special issue. We hope that this will provide both an informative guide to contemporary crystallographic data analysis and a benchmark reference for the *CCP*4 suite in its current form. We also gratefully thank the administrative staff of CCP4 for their essential support in organizing the meeting, and Charles Ballard for his role as guest editor of these proceedings.

